# Local Treatment of Hepatocellular Carcinoma with Oligometastases: A Systematic Review and Meta-Analysis

**DOI:** 10.3390/cancers15133467

**Published:** 2023-07-02

**Authors:** Sooyeon Kim, Jungsue Lee, Chai Hong Rim

**Affiliations:** 1 College of Medicine, Korea University, Seoul 02841, Republic of Korea; sykim4210@korea.ac.kr (S.K.); sueannie@korea.ac.kr (J.L.); 2 Department of Radiation Oncology, Korea University Ansan Hospital, Ansan 15355, Republic of Korea

**Keywords:** hepatocellular carcinoma, oligometastases, radiotherapy, radiofrequency ablation

## Abstract

**Simple Summary:**

Oligometastasis is defined as a malignancy disease characterized by a limited number of metastatic foci (3–5). In studies of lung cancer and prostate cancer, local treatment such as radiotherapy or surgery increased the prognosis of oligometastases. The efficacy of local treatment for oligometastasis of hepatocellular carcinoma is not well known. This meta-analysis reports that active local treatment of oligometastases in hepatocellular carcinoma significantly increased survival rates and improved survival outcomes.

**Abstract:**

Although recent studies have shown favorable results after local treatment for oligometastases, the clinical decision of applying local treatment for oligometastatic hepatocellular carcinoma (HCC) remains controversial. This meta-analysis aimed to investigate the benefits of local treatment for HCC oligometastases. Pubmed, Embase, Medline, and the Cochrane library were searched for studies until 1 May 2022. Clinical studies involving at least five cases of HCC oligometsatases treated with local modalities were included. The primary endpoint was overall survival (OS). The benefit of local treatment was assessed as the pooled odds ratio (OR) among comparative series, and the pooled OS percentile was calculated from all studies including patients treated with local treatment. Complications of grade ≥ 3 were assessed subjectively. A total of 10 studies involving 527 patients were included. Radiotherapy and radiofrequency ablation (RFA) were mainly performed (six and five studies) as local modalities treating oligometastases. Pooled OR of comparative series favored the use of local treatment (4.664, 95% confidence interval [CI]: 2.595–8.380, *p* < 0.001, I^2^: ~0.0%). Including all cohorts with patients who underwent local treatment, pooled rates of 1-year OS were 71.8% (95% CI: 59.0–81.9; I^2^ = 81.5%), and pooled 2-year OS were 43.3% (95% CI: 29.1–59.6; I^2^ = 85.4%). Except for temporal or pre-existing toxicities, grade ≤ 3 complications were reported less than 10% in most studies, although common toxicities include pneumothorax and hematologic deficiency after RFA and radiotherapy, respectively. Grade 5 toxicity has not yet been reported. This systematic review supports the application of local treatment for treating HCC oligometastases.

## 1. Introduction

Traditional oncological principles have focused on systemic chemotherapy as the main treatment approach for metastatic cancer, assuming that metastasis occurs after the hematogenous spread of tumor cells from the primary site. Nevertheless, throughout the 20th century, many researchers have actively performed metastasis-directed treatment in patients with a limited disease burden. Pastorino et al. [1] reported the outcome of surgery on 5206 patients with lung metastases, indicating a 5-year survival rate of 33% after complete resection. In a British multicenter study of 1422 patients [2], stereotactic radiotherapy yielded a 2-year survival rate of 79.2% for patients with a limited number of metastatic lesions. These clinical results support the existence of an intermediate stage before localized cancer progresses to disseminated disease. This stage was named oligometastasis by Hellman and Weischelbaum [3], and studies are being actively conducted to evaluate the benefit of local modalities for its treatment. The oncological benefits of local treatment for oligometastasis include metastatic cascade arrest, elimination of disease sources, and symptomatic relief [4].

Before mid-2010, mostly single-arm oligometastasis clinical studies have been reported with ‘better than previously expected’ outcomes [5]. However, in recent years, several randomized studies have reported the benefits of local treatment for oligometastatic lung and prostate cancers [6,7,8,9,10], suggesting an increase in the clinical application of local treatment for oligometastases. The number of clinical studies on oligometastasis in HCC is relatively small compared to that of other cancer types (9). Such scarcity of literature is because HCC is a rare disease in Western countries, and life expectancy after extrahepatic metastasis is considered to be short [11,12]. Nonetheless, pioneering researchers from East Asia have attempted active local treatment for oligometastasis in HCC. Recent technical development of locoregional modalities, such as superselective transarterial therapy, local thermal ablation with CT-guidance or using microwave, and stereotactic radiotherapy, have increased the feasibility of local treatment for oligometastatic HCC [13,14,15]. In a propensity-matching study including patients with HCC and oligometastatic lymph node metastases, Pan et al. [16] reported that the survival rate of the group that underwent radiofrequency ablation (RFA) was significantly higher than that of the control group (1-year overall survival [OS], 58.3% vs. 17.9%). In another propensity-matching study by Kim et al. [17] involving patients with HCC and lung oligometastases, the survival rate was significantly higher in the group where the local modality was combined with systemic treatment, compared with the group in which only systemic treatment was performed (2-year OS 66.6% vs. 31.3%).

Although systemic treatment is the current standard for metastatic HCC, sorafenib showed only moderate oncological benefits [18], and the atezolizumab-bevacizumab combination has not yet been widely adopted despite its superior efficacy compared with the previous standard [19]. Regarding local treatment for oligometastatic HCC, clinical decisions are made based on the limited available literature references without consensus. Therefore, this systematic review and meta-analysis aimed to evaluate the oncological efficacy of local treatment for oligometastatic HCC.

## 2. Methods

### 2.1. Inclusion Criteria and Search Strategy

This study followed the PRIMSA guidelines, and the Cochrane Handbook was referred to for the methodological approach. The research hypothesis was formulated as PICO questions: “Is local treatment efficient for the survival of patients with oligometastatic HCC compared with the previous standard methods?” The previous standard treatment referred to sorafenib or supportive care based on the study period of candidate studies. This study was designed to investigate the effectiveness and feasibility of local treatment for HCC oligometastases. We used the search terms “oligomet*” OR “limited metastases” AND “hepatocellular” OR “hcc” to search databases, including PubMed, Embase, Medline, and the Cochrane Library for available studies up to 1 May 2022. Clinical studies, including randomized trials and non-randomized studies (case-control studies, cohort studies, and case-series) were included if they fulfilled the following inclusion criteria: (1) involved at least five cases of HCC oligometastases treated with local modalities, including surgery, radiotherapy, and radiofrequency ablation; and (2) included overall survival (OS) as the primary endpoint in the analysis. The following types of studies were excluded: (1) case reports, lab studies, systematic reviews, letters, editorials, and national database studies; (2) studies that did not investigate efficacy of local modalities (e.g., systemic treatment only); and (3) studies without OS data. Conference abstracts were included if they fulfilled the inclusion criteria. Language restrictions were not applied, and external language consultations were performed where necessary. Multiple studies from a single institution were included if they did not have overlapping data. However, in case of overlapping data, we included either a study with a larger number of patients or the latest study with a similarly large number of patients.

### 2.2. Data Collection

We used a pre-designed sheet to collect general information, including author name, publication, patient recruitment periods, study design, and conflicts of interest. Subsequently, we collected clinical information, including the number of patients, modality of local treatment, organ of metastases, number of metastases, Child-Pugh class, rates of OS, clinical factors affecting OS, and complications of grade ≥ 3. Three independent reviewers performed the study selection and data collection, and any disagreement was resolved by re-evaluating the literature.

### 2.3. Quality Assessment

Since the majority of studies found during preliminary searches were non-randomized, we used the Newcastle–Ottawa scale [20], which is recommended by the Cochrane handbook for the assessment of observational studies [21]. Studies with a score of 8–9, 6–7, and ≤5 were evaluated as high, medium, and low-quality studies, respectively. A detailed scoring sheet and subjective notes from the investigator are both provided. Following the guidance provided in the Cochrane handbook, which suggests that observational studies with a high risk of bias are not recommended for meta-analysis, low quality studies were excluded from further meta-analysis with the consent of the authors.

### 2.4. Effect Measures and Data Synthesis

The primary endpoint of the present study was OS. The effect measure used was the odds ratio (OR) of OS difference with the application of local treatment, including comparative studies, and the pooled percentile of OS rates from both comparative and single-arm studies. To calculate OR, the 1-year OS rate of comparative series was used, taking into account the range of median OS (7–18 months) based on preliminary literature analyses [16,17,22]. Grade ≥ 3 complication rates were acquired and subjectively reviewed, considering the heterogeneity of treatment modalities and assessment of toxicities. Considering the possible heterogeneity among treatment strategies of institutions, and referencing the Cochrane handbook that the random effects model should be used to integrate results from observational studies [21], the random effects model was used for pooled estimations.

For heterogeneity assessment, Cochran’s Q [23] test and I^2^ statistics [24] were performed. I^2^ statistics of 25%, 50%, and 75% were considered as low, moderate, and high heterogeneity, respectively. Publication bias was evaluated using a visual assessment of the funnel plot and quantitative Egger’s test. If possible, publication bias was noted with a two-tailed *p*-value of <0.1 in the Egger’s test with significant visual asymmetry in the funnel plot. Duval and Tweedie’s trim and fill method [25] was used to obtain adjusted estimates. All statistical analyses were performed using Comprehensive Meta-Analysis version 3 (Biostat Inc., Englewood, NJ, USA).

## 3. Results

### 3.1. Study Selection and Characteristics

**Figure 1:**
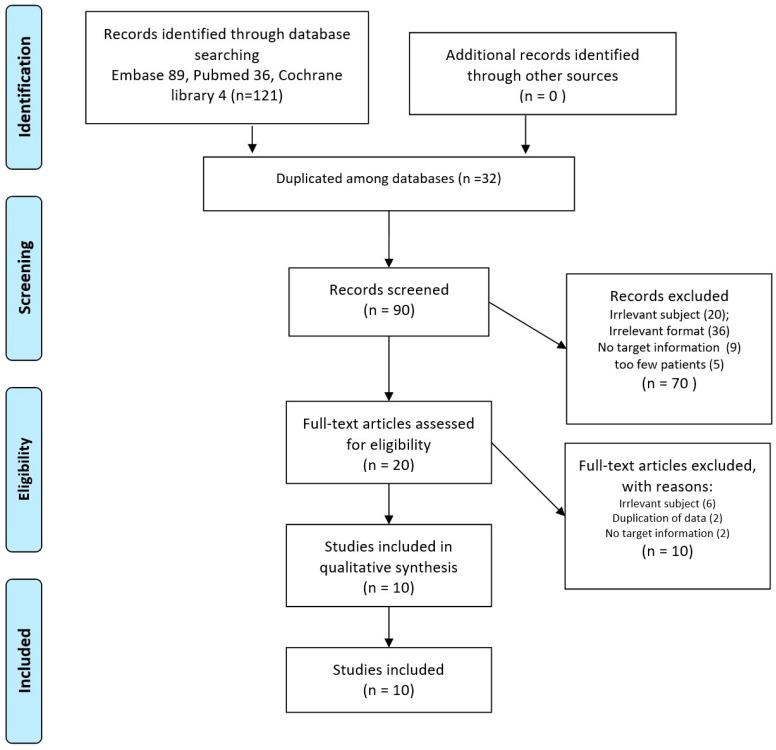
The inclusion process.

Of 121 initially identified studies, 32 were excluded due to duplication among databases. After the abstract screening of 90 studies, 70 were excluded due to irrelevant subjects or formats, no target information, or a low number of included patients. A full-text review was performed on 20 studies, and based on the inclusion criteria, 10 studies [16,17,22,26,27,28,29,30,31,32] involving 527 patients were finally included in the present study. Figure 1 illustrates the selection process of studies.

All included studies were written in English and were published in Korea (n = 4), China or Taiwan (n = 4), and Japan (n = 2). The earliest and latest studies recruited patients from 1992–2019 and 2014–2017, respectively. Three studies were designed to have comparative arms (e.g., patients who received RFA vs. those who did not receive RFA) using propensity score methods, whereas others were single-arm case-series studies. Six and five studies employed radiotherapy and RFA, respectively, as local modalities for treating HCC oligometastases. Furthermore, six and three studies demonstrated treatment of lung and lymph node metastases, respectively, while bone and adrenal metastasis treatment was demonstrated in one study each. Table 1 shows the general characteristics of the included studies.

### 3.2. Risk of Bias Assessment

Publication bias assessment was performed while analyzing 1-year and 2-year OS percentiles. In the 1-year survival analysis, Egger’s test *p*-value was 0.04015; however, the trimmed value using Duval and Tweedie’s method was not adjusted from the original value. No significant publication bias was observed in the 2-year OS analysis. Supplementary Figure S1 shows funnel plots and results of the publication bias assessment. Regarding the quality assessment of included studies using the Newcastle-Ottawa scale, it should be noted that the studies included in this systematic review focused specifically on HCC oligometastasis and were conducted by institutions at the tertiary hospital level. No study reported follow-up losses that could induce significant bias. Therefore, all studies were scored except for queries related to comparability, selection of the non-exposed cohort, and the follow-up period. Propensity matching studies accounting for two or more clinical factors were fully scored for comparability. As a result, all three matching studies scored 2 points, while other single arm studies did not. The selection of the non-exposed cohort query was not applicable to single-arm studies. Considering the expected survival of oligometastatic HCC patients, studies with a median follow-up period of ≥1 year scored a point. Therefore, in quality assessment using the Newcastle-Ottawa scale, three comparative studies scored 8–9, whereas all single-arm studies scored 6–7. Since all included studies were considered to be of moderate or high quality, they were included in the pooled analyses. Detailed scoring results are shown in Supplement Data S1.

### 3.3. Clinical Information and Synthesized Results

The median follow-up period ranged 12–28 months (median value: 17.8 months). The proportion of Child-Pugh A class patients ranged 33.3%–100% (median value: 94.9%). The median OS ranged 9.8–33.5 (median value: 19.2 months) including cohorts treated with local modalities. The median survival period of the all cohorts ranged 7.4–33.5 months (median value: 17.4 months). Among the clinical factors affecting OS, alpha-fetoprotein (AFP) levels (three studies), Child-Pugh class (four studies), application of local treatment (three studies), and controlled primary disease (three studies) were commonly reported. Table 2 summarizes the clinical information of the included studies.

**Figure 2:**
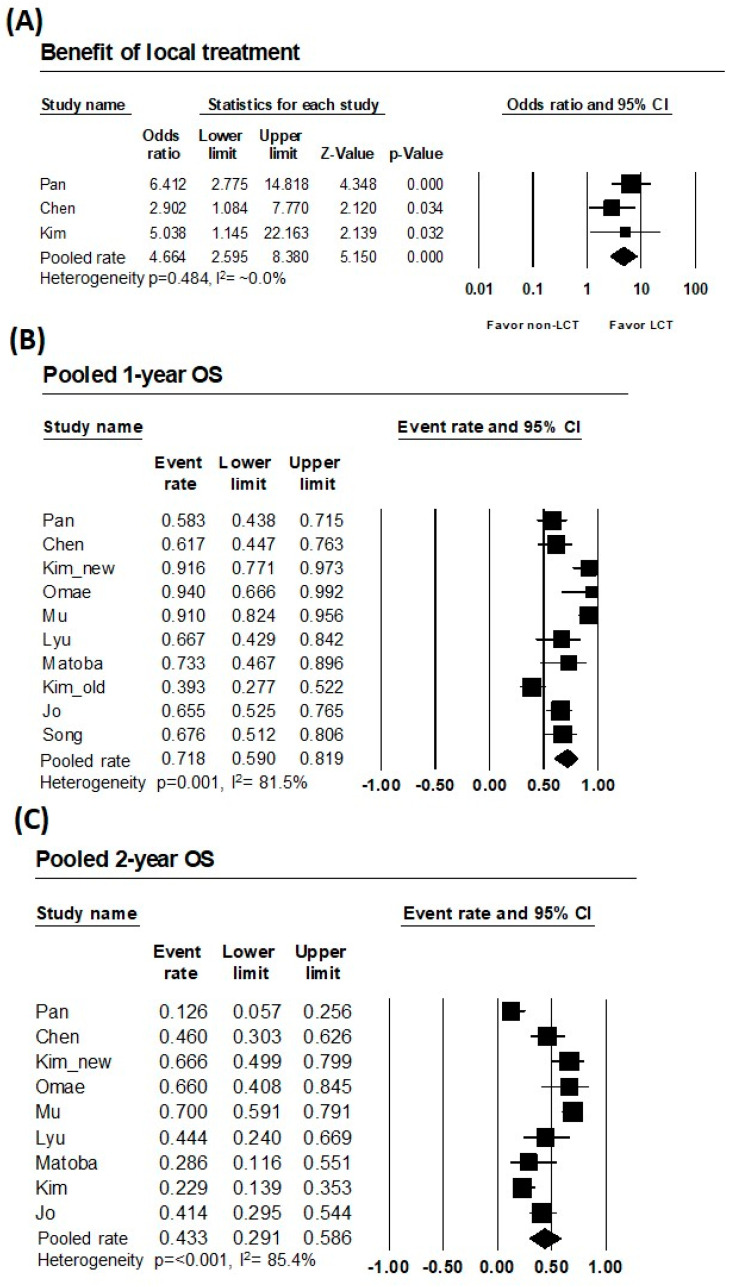
Outcomes of pooled analyses. Forest plots of the (**A**) pooled odds ratio of comparative series, (**B**) 1-year, and (**C**) 2-year pooled overall survival percentiles of cohorts treated with local modalities.

All three comparative studies reported the benefits of local treatment in terms of OS. Pooled analysis of comparative studies yielded an OR of 4.664 (95% confidence interval [CI]: 2.595–8.380, *p* < 0.001; heterogeneity: 0.484, I^2^ ~ 0.0%, very low). For the studies where patients who underwent local treatment were included, pooled rates of 1-year and 2-year OS were 71.8% (95% CI: 59.0–81.9; heterogeneity *p* = 0.001, I^2^ = 81.5%) and 43.3% (95% CI: 29.1–59.6; heterogeneity *p* < 0.001, I^2^ = 85.4%), respectively. Table 3 presents the pooled results, and these results are presented as forest plots in Figure 2.

Grade 5 complications due to local treatment have not yet been reported. Among RFA studies, Mu et al. [30] reported a pneumothorax rate of 9.5% necessitating chest tube insertion, whereas Lyu et al. [28] reported grade 3 hypertension in eight out of 33 sessions (24.2%). However, it is important to note that such hypertension was temporary, and all cases were controlled and restored after rest or administration of calcium channel blockers. Among radiotherapy studies, hematologic complications were the most commonly observed serious complications. Jo et al. [26] reported three cases of grade 4 leukopenia (5.2%) and one case of grade 3 leukopenia (1.7%) after stereotactic body radiotherapy (SBRT). Notably, all grade 4 leukopenia cases had grade 3 leukopenia before SBRT. Table 2 shows the details of the toxicities reported.

## 4. Discussion

The prognosis of metastatic HCC is generally poor, and limited effective systemic treatment options are available apart from sorafenib. Aino et al. [12] from Kurume University reported the course of 419 cases of untreated HCC with extrahepatic metastases, revealing a median survival of 6.8 months and a one-year survival rate of 31.6%. According to an Italian multicenter study by Gianni et al. [33], the median survival of patients with untreated BCLC C and D HCC was seven and six months, respectively. In an Egyptian study, the median survival time of patients with untreated HCC was only 2.3 months [34]. According to landmark studies, although sorafenib administration provided a survival gain of 2.3–2.8 months, patients with extrahepatic metastasis did not benefit from sorafenib in subgroup analyses [18,35]. Moreover, while the atezolizumab-bevacizumab combination showed favorable results compared with sorafenib, it is not cost-effective, precluding global availability [19]. Therefore, additional modalities to enhance the outcomes of patients with metastatic HCC are necessary.

The studies included in the present meta-analysis were conducted before the atezolizumab-bevacizumab combination was approved for treatment [36]. Moreover, sorafenib was administered to only 13–36% of patients in the included studies. Hence, the OS rates (1-year and 2-year OS rates: 71.8% and 43.3%, respectively) obtained in this meta-analysis are encouraging. Although randomized studies are yet to be published, pooled analyses of propensity matching series showed a significant OR of 4.664 with very low heterogeneity, supporting the benefit of local treatment for metastatic HCC.

The role of local treatment in oligometastasis has recently been highlighted. Oligometastasis is defined as a disease with equal or fewer than three or five metastatic foci, or a disease extent that can be covered by local treatments [37]. Notably, recent randomized studies have demonstrated that the application of local modalities has a valid oncological benefit in lung and prostate cancers with oligometastasis [6,7,8,9]. However, clinical decisions regarding local treatment for oligometastasis of HCC are controversial due to the absence of randomized or large-scale comparative studies. Our meta-analysis provides evidence suggesting the possibility of significantly improving prognosis through the application of local treatment for oligometastases. As far as we know, this study is the first meta-analysis on the efficacy of local treatment of HCC oligometastases. The findings from this study can be valuable in determining treatment strategies for such incurable situations.

When treating patients with oligometastatic HCC, efficacy and feasibility of local treatment and available systemic treatment options should be considered. Based on favorable results of analyses regarding both pooled OR and survival rates, the present study supports the application of local treatment for oligometastasis of HCC. As per the studies included in the present meta-analysis, patients with low AFP and controlled primary disease may have a low amount of dormant metastases and less disease progression probability, and hence, the application of local treatment should be actively considered [16,17,22,27]. Moreover, liver function, commonly evaluated in the Child-Pugh class, has been reported as a significant factor for OS in several studies included [26,28,29,30]. Therefore, local treatment should be considered for patients with oligometastatic HCC and Child-Pugh class A or comparable liver function. Patients with advanced HCC might not be indicated for surgery due to liver decompensation [38]; however, non-invasive ablative modalities, such as SBRT or RFA, could be a viable option for treating oligometastatic HCC, as demonstrated in studies included in the present systematic review. In addition, SBRT may be considered for areas where RFA is difficult to perform, in order to reduce the possibility of mechanical complications (i.e., pneumothorax) associated with RFA [39]. In patients with poor hematologic reserves, the range of the liver or bone marrow included in the dose distribution should be reviewed while applying radiotherapy [40].

Further investigation is warranted to evaluate the efficacy of systemic treatment and the combination of systemic and local treatments for managing oligometastatic HCC. Subgroup analyses of sorafenib landmark studies did not demonstrate significant survival benefit in patients with extrahepatic spread [18,35]. In studies proving the effectiveness of atezolizumab-bevacizumab combination, patients with extrahepatic spread were not analyzed separately [36]. However, according to a recent meta-analysis, combination therapy consisting of sorafenib and radiotherapy was safe and achieved superior results compared with monotherapy [41]. The combination of local treatments, including RFA or radiotherapy with sorafenib is currently being actively studied (NCT01328223, NCT01319942, NCT03535259, and NCT01470495). In the future, the efficacy of atezolizumab-bevacizumab in combination with local modalities for treating oligometastatic HCC should be studied.

This study has several limitations that should be acknowledged. Meta-analyses, including observational studies are controversial due to inherent heterogeneity between studies and uncontrolled confounding variables, which can affect pooled estimates [42]. However, not all clinical decisions in the field of oncology are supported by randomized studies. For intractable diseases lacking established standard treatment, such as oligometastatic HCC, a meta-analysis of observational studies may be one of the few available strategies to determine possible therapeutic options [43,44]. Analyses of heterogeneity, quality of studies, publication biases, and subgroups were performed to increase the reliability of this study. One limitation of our meta-analysis is the small number of included comparative studies. Nevertheless, the low heterogeneity observed among the studies supports the significance of local treatment for oligometastatic HCC. The pooled results showed favorable OS among patient cohorts who underwent local treatment, aligning with our hypothesis. In addition, although heterogeneity was low for the OR (i.e., the main measurement used to assess the benefit of local modalities), heterogeneity was observed in the pooled analyses of OS percentile. In HCC oligometastases, various factors, such as disease burden, liver function, metastatic site, AFP, and type of local modalities can affect the OS. As more futures studies are published, the OS of specific subgroups will need to be evaluated by performing a narrower subject analysis while considering these clinical factors.

## 5. Conclusions

The oncologic benefit was shown consistently in the pooled analysis and the individual comparative series, and the pooled survival result was favorable as compared to that of the previous literature. Apart from temporal or pre-existing toxicities, grade ≥ 3 complications were less than 10% in most studies. Overall, this systematic review supports the application of local modalities for the treatment of oligometastatic HCC.

## Figures and Tables

**Table 1:** Characteristics of the included studies.

Author	No. of Patients	Affiliation	Study Design	Recruiting Year	Metastatic Organ	OM No.	OM Definition	CPC A (%)	Other Metastasis (%)
Pan	46 (RFA)	Sun Yats sen center, Taiwan	PSM Retrospective cohort	2004–2013	lymph node	2.22 ± 1.35	NA	95.7	26.1
	73 (non-RFA)					2.74 ± 1.37		93.5	37.0
Chen	34 (combined)	Sun Yat sen center, China	PSM Retrospective cohort	2013–2016	lung		≤5	94.1	17.6
	34 (sorafenib alone)							88.2	17.6
Kim	36 (combination of local, systemic therapies)	Yonsei University College of Medicine, Korea	PSM Retrospective cohort	2008–2015	lung		≤4	100.0	
	22 (systemic therapy alone)							100.0	
Omae	16	Okayama University Medical School, Japan	Case series	2001–2013	lung	single 43.8%	≤5		37.5
Mu	79	Sun Yat sen center, China	Case series	2004–2015	lung 56%; lymph node 13%	single 67.1%	≤3	98.7	15.2
Lyu	18	Sun Yat sen center, China	Case series	2004–2015	adrenal		≤2		
Matoba	15	Kanazawa Medical University, Japan	Case series	2014–2017	lymph node	single 66.7%	≤5	33.3	
Kim	59	Yonsei University College of Medicine, Korea	Case series	1992–2019	bone	≤5 lesions	≤5		
Jo	58	9 centers, Korea	Case series	2011–2018	lung		NA		
Song	37	Asan hospital, Korea	Case series	2006–2011	lung		≤5		

Abbreviations: OM, oligometastases; CPC, Child-Pugh class; RFA, radiofrequency ablation; PSM, propensity score matching; NA, not accessible.

**Table 2:** Treatment profiles and clinical outcomes.

Author	No. of Patients	Study Design	Treatment Method	Primary Controlled	Follow-Up Period	Overall Survival (M, Median)	Factors Affecting Survival	Toxicity (Grade ≥ 3)
Pan	46 (RFA)	PSM Retrospective cohort	RFA (sorafenib 26%) vs. non-RFA (sorafenib 20%)	54.4%	14	M13.0 months 1/2y: 58.3/12.6 (*p* = 0.001)	Controlled primary disease, Local treatment, Other metastasis	No grade ≥ 3 toxicity
	73 (non-RFA)			50.0%	13.8	M7.8 months 1/2y: 17.9/0 (*p* = 0.001)		
Chen	34 (combined)	PSM Retrospective cohort	sorafenib & local therapies (TACE, RFA, ^125^I) vs. sorafenib	35.3%		M18.4 months 1/2y: 61.7/46%	AFP, Local treatment, Macrovascular invasion	
	34 (sorafenib alone)			26.5%		M7.4 months 1/2y: 35.7/23.7% (*p* = 0.015)		
Kim	36 (combination of local, systemic therapies)	PSM Retrospective cohort	Local (surgery or RT) vs. systemic therapies			1/2y: 91.6/66.6% (*p* < 0.001)	AFP, Local treatment	1 case (2.8%) of grade 3 pneumonitis after surgery
	22 (systemic therapy alone)					1/2y: 68.4/31.2% (*p* < 0.001)		No grade ≥ 3 toxicity
Omae	16	Case series	RFA (systemic therapy 36%)	25.0%	26	M26 months 1/2y: 94/66% (*p* = 0.85)		No mortality related to RFA
Mu	79	Case series	RFA (sorafenib 17.7%)		28	M33.5 months 1/2y: 91/70%	AFP (*p* = 0.054), CPC, Time to metastasis	9 cases of pneumothorax (9.5%, 9/95) necessitate chest tube insertion
Lyu	18	Case series	RFA		17~18	M21.8 months 0.5/1/2y: 88.9, 66.7, 44.4% (*p* = 0.043)	CPC	8 of 33 sessions of grade 3 hypertension, which was restored after treatment) No grade ≥ 4 toxicity
Matoba	15	Case series	SBRT (sorafenib 13.3%)		18.1	1/2y: 73.3/28.6%	CPC (univariate analysis)	No grade ≥ 3 toxicity
Kim	59	Case series	HFRT		12	MOS 9.8 mo 1/2y: 39.3, 22.9% (*p* < 0.001)	Controlled primary disease, Extraosseous metastasis	
Jo	58	Case series	SBRT (chemotherapy, 36.2%)			MOS 16.3 months (1/2y: 65.5, 41.4%) MPFS 4.9 months (1/2y: 22.4, 13.5%)	CPC, Controlled primary disease, RT response	3 cases of grade 4 Leukopenia (5.2%); 2 cases of grade 3 pneumonia (3.4%), 1 case of leukopenia (1.7%), 1 case of dermatitis (1.7%)
Song	37	Case series	SBRT		19.9	M19.9 months 1/3y: 67.6/35.5	Treatment modality	No significant RT complication

Abbreviations: RFA, radiofrequency ablation; NRCT, non-randomized controlled study; PSM, propensity score matching; TACE, transarterial chemoembolization; RT, radiotherapy; SBRT, stereotactic body radiotherapy; HFRT, hypofractionated radiotherapy, AFP, alphafetoprotein, CPC, Child-Pugh class, OS, overall survival; PFS, progression-free survival.

**Table 3:** Pooled rates of synthesized results.

Subject	No. of Studies	No. of Cases	Heterogeneity *p*	I^2^ (%)	Heterogeneity Assessment	Pooled Rate (95% CI)
Comparative OR	3	245	0.484	~0.0%	Very low	4.664 (2.595–8.380)
1-year OS	10	398	0.001	81.5%	High	71.8% (59.0–81.9)
2-year OS	9	361	<0.001	85.4%	High	43.3% (29.1–58.6)

Abbreviations: CI, confidence interval; OR, odds ratio; OS, overall survival.

## Data Availability

The data presented in this study are available in this article and supplementary material.

## Supplementary Materials

The following supporting information can be downloaded at: https://www.mdpi.com/article/10.3390/cancers15133467/s1. Figure S1: Funnel plot assessing publication bias in analaysis of 1- and 2-year survival; Data S1: Scoring sheet according to New-Castle Ottawa scale.
