# Supplementary material for: Local Treatment of Hepatocellular Carcinoma with Oligometastases: A Systematic Review and Meta-Analysis
Source: Cancers (Basel). 2023 Jul 2;15(13):3467. doi: 10.3390/cancers15133467 (PMC10340244; doi:10.3390/cancers15133467)
Supplement: Supplementary file 1 [file cancers-15-03467-s001.zip › cancers-2467460-supplementary.pdf]

Supplement Figure S1A. Funnel plot assessing publication bias in analaysis of 1-year survival

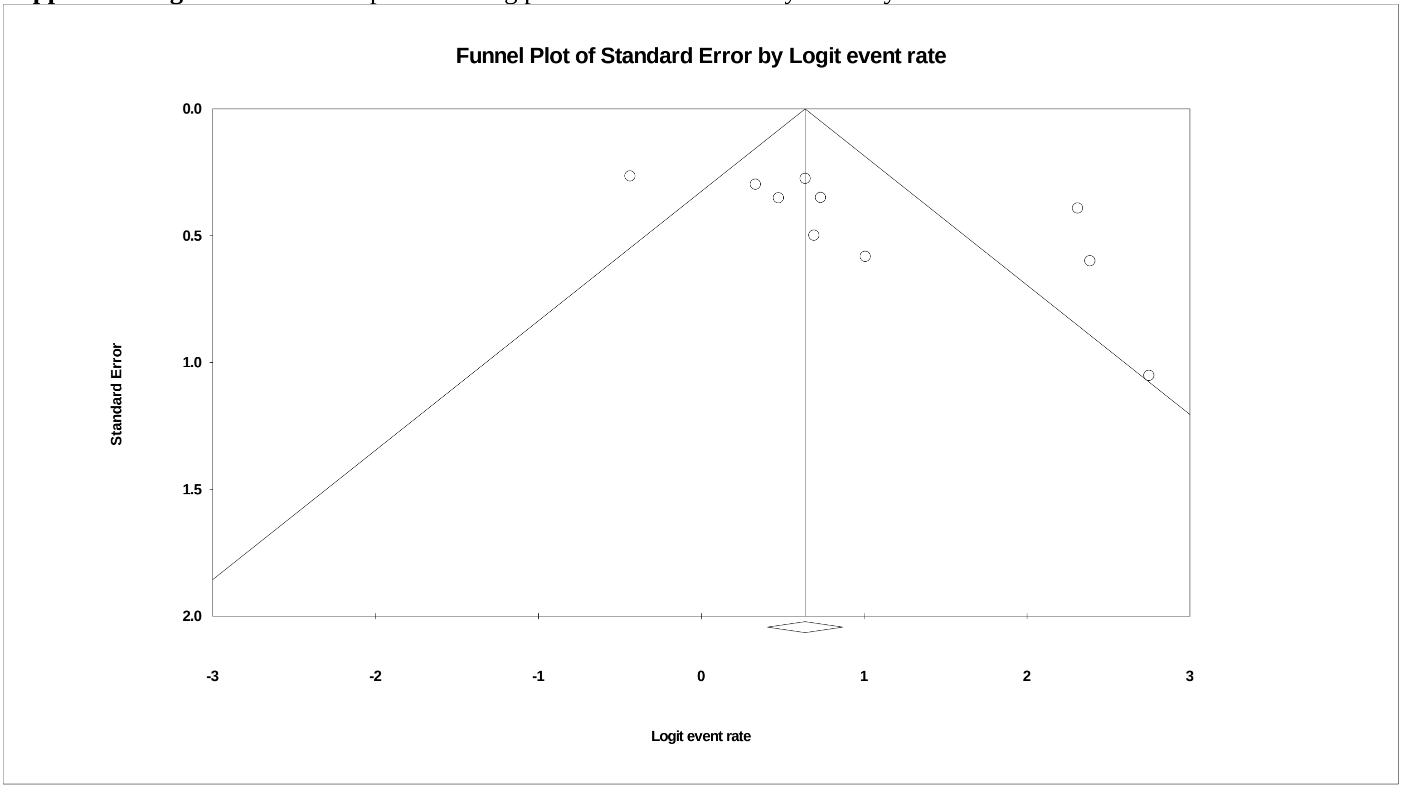

Egger's test p value: 0.04015
Trim and fill value: 71.8% (same as the original value)

Supplement Figure S1B. Funnel plot assessing publication bias in analysis of 2-year survival

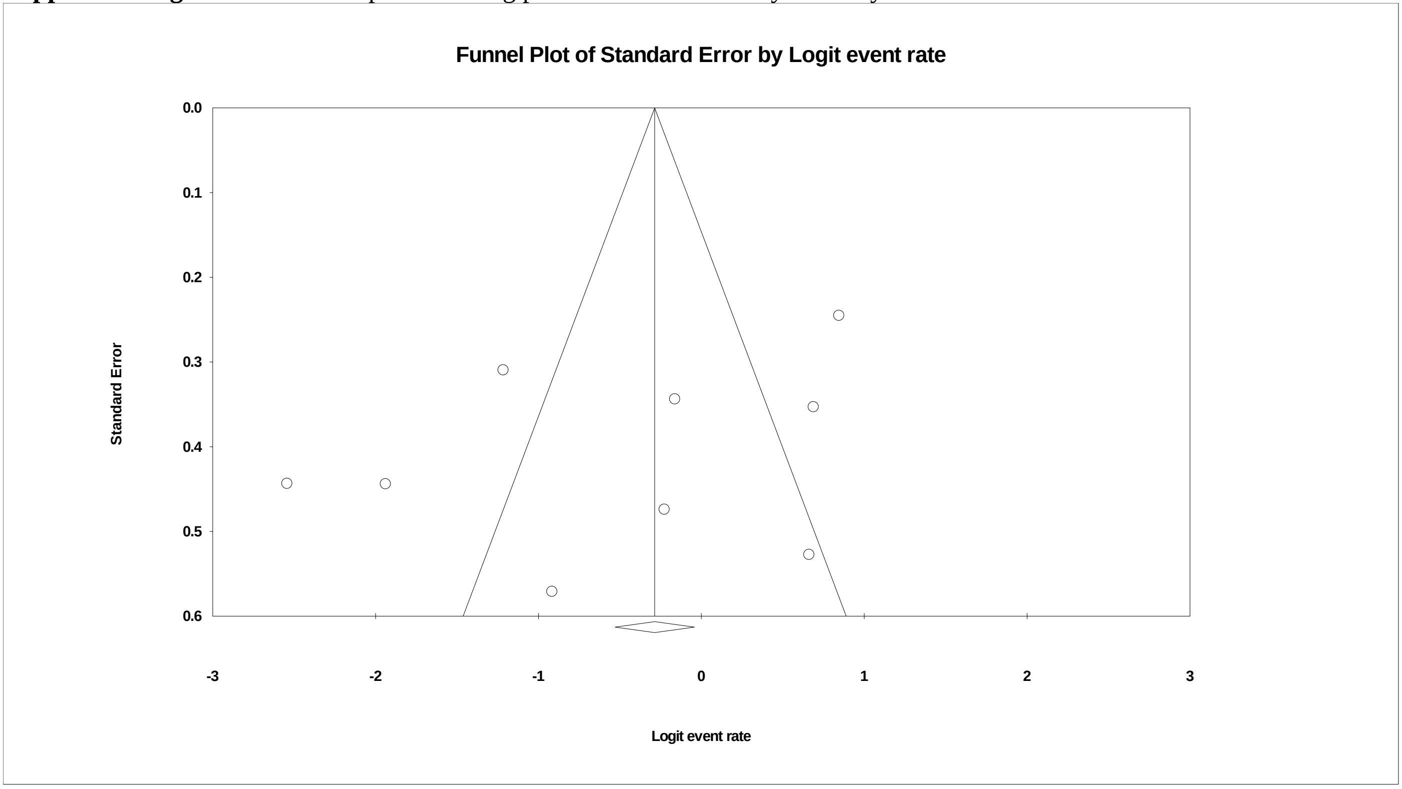

Egger's test p value: 0.22250

Supplement Data S1. Scoring sheet according to New-Castle Ottawa scale

	Selection				Comparability	Outcome			**Overall score (9 to be full)**
	1	2	3	4	1	1	2	3	
	Representativeness of the exposed cohort	Selection of the non exposed cohort	Ascertainment of exposure	Outcome of interest was not present at start of study	Comparability of cohorts on the basis of the design or analysis	Assessment of outcome	Was follow-up long enough for outcomes to occur	Adequacy of follow up of cohorts	
Pan	1	1	1	1	2	1	1	1	9
Chen	1	1	1	1	2	1	0	1	8
Kim	1	1	1	1	2	1	0	1	8
Omae	1	0	1	1	0	1	1	1	6
Mu	1	0	1	1	0	1	1	1	6
Lyu	1	0	1	1	0	1	1	1	6
Matoba	1	0	1	1	0	1	1	1	6
Kim	1	0	1	1	0	1	1	1	6
Jo	1	0	1	1	0	1	1	1	6
Song	1	0	1	1	0	1	1	1	6

The studies included in this systematic review investigated a narrow range of diseases called HCC oligometastasis and were conducted by institutions at the level of tertiary hospitals. No study reported follow-up losses which could induce a significant bias. Therefore all studies were scored except for queries related to comparability, selection of the non-exposed cohort, and the follow-up period. Propensity matching studies corrected for two or more clinical factors were fully scored for comparability; therefore all three matching studies scored 2 points and other single arm studies did not. Single-arm studies did not scored the selection of the non-exposed cohort query. Considering the expected survival of oligometastatic HCC patients, studies with a median follow-up period of ≥ 1 year scored a point.
